# On the Existence of Wavelet Symmetries in Archaea DNA

**DOI:** 10.1155/2012/673934

**Published:** 2011-11-28

**Authors:** Carlo Cattani

**Affiliations:** Department of Mathematics, University of Salerno, Via Ponte Don Melillo, 84084 Fisciano, Italy

## Abstract

This paper deals with the complex unit roots representation of archea DNA sequences and the analysis of symmetries in the wavelet coefficients of the digitalized sequence. It is shown that even for extremophile archaea, the distribution of nucleotides has to fulfill some (mathematical) constraints in such a way that the wavelet coefficients are symmetrically distributed, with respect to the nucleotides distribution.

## 1. Introduction

In some recent papers the existence of symmetries in nucleotide distribution has been studied for several living organisms [1–6] including mammals, fungi [1–4], and viruses [5, 6]. Thus showing that any (investigated) DNA sequence, when converted into a digital sequence, features some fractal shape of its DNA walk and an apparently random-like distribution. However, when the short wavelet transform maps the digital sequence into the space of wavelet coefficients, and these coefficients are clustered then they are located along some symmetrical shapes.

One of the main tasks of this paper is to show that although the distribution of nucleotide, in any DNA sequence, can be considered as randomly given, when we compare a random sequence (and the corresponding random walk) with a DNA sequence (and walk) it can be seen that there exists some distinctions. So that the nucleotides distribution seems to side with a random distribution with some constraints. These constraints (rules) are singled out in the following, by showing the existence of hidden geometry which underlies the structure of a DNA sequence.

In other words, nucleotides are distributed along any DNA sequence at first apparently randomly but at second analysis according to some (statistical) mathematical constraints which does not allow a given nucleotide to be arbitrarily followed by any other remaining nucleotides.

It is interesting to notice that even in the primitives organisms which billions of years ago have been colonizing the earth under extreme conditions of life, their DNA has to fulfill the same constraints of the more evolved DNAs.

In order to achieve this goal some fundamental steps have to be taken into consideration and discussed.

1. Since DNA is a sequence of symbols, a map of these symbols into numbers has to be defined. In the following we will consider the complex unit roots map, which has the advantage of being unitary and distributed along the unit circle.
2. The indicator matrix is defined on the the indicator map. This matrix is important in order to draw the dot plot of the DNA sequence and from this plot we can see that apparently nucleotides seem to be randomly distributed. However, we will show by wavelet analysis that they look randomly distributed, while they are not.
3. The Ulam spiral adapted to DNA sequences is defined in order to single out some geometrical patterns.
4. Random walks on DNA, or short DNA walks, show that the random walks look like fractals.
5. The analysis of clusters of wavelet coefficients show that DNA walks have to fulfill some geometrical constraints.

In all DNA sequences, analyzed so far, for different kinds of living organisms, this geometrical symmetry [1–6] has been detected. In the following this analysis is extended also to archaea, since they might be considered at the early stage of life and their DNA is compared with more evolved microorganisms as bacteria.

It will be shown that, inspite of the many similarities with random sequences, only the wavelet analysis makes it possible to single out some distinctions. In particular, the wavelet coefficients of all (analyzed) organisms tend to fulfill a minimum principle for the energy of the signal. Also the archaea which often live in extreme environments have to fulfill the same geometrical rule of any other living organism.

The analysis of DNA by wavelets [7–9], as seen in [8–12], helps to single out local behavior and singularities [7, 13] or to express the scale invariance of coefficients [14]. Also multifractal nature of the time series [15–17] can be easily detected by wavelet analysis.

Some previous paper have studied various sequences of DNA such as leukemia tet variants, influenza viruses such as the A (H1N1) variant, mammalian, and a fungus (see [1–3, 14]) provided by the National Center for Biotechnology Information [18–21]. In all these papers it was observed that DNA has to fulfill not only some chemical steady state given by the chemical ligands but also some symmetrical distribution of nucleotide along the sequence. In other words, base pairs have to be placed exactly in some positions.

According to previous results, it will be shown that as any other living organisms also these elementary organisms have DNA walks with fractal shape and wavelet coefficients bounded on a short-range wavelet transform. In other words, also anaerobic organism which should be understood as the most elementary at the first step of life have the same symmetries on wavelet coefficients as for more evolved organism, so that life has to fulfill some constrained distribution of nucleotides in order to give rise to some organism even at the most elementary step.

In particular, in Section 2, some remarks about the analysed data are given.  Section 3 deals with some elementary plots which can easily visualize the distribution of nucleotides. The Ulam spiral plot is also proposed for the first time and it is observed a different distribution of weak/strong hydrogen bonds.  Section 4 provides some definitions about parameters of complexity. We will notice that all these parameters give rise to the same classification of organism.  Section 4 proposes a complex numerical representation of DNA chains and random walks, while in final Section 6 the short wavelet trasform is given in order to single out some symmetries at the lower order of transform.

## 2. Materials and Methods

In the following we will take into consideration some genome, complete sequences of DNA, concerning the following archaea:

**h1**: *Aeropyrum pernix* K1, complete genome. DNA, circular, 1669696 bp, [18–21], accession BA000002.3. Lineage: *Archaea*; Crenarchaeota; Thermoprotei; Desulfurococcales; Desulfurococcaceae; Aeropyrum; *Aeropyrum pernix*; *Aeropyrum pernix* K1. This organism, which was the first strictly aerobic hyperthermophilic archaeon sequenced, was isolated from sulfuric gases in Kodakara-Jima Island, Japan in 1993.
**h2**: *Acidianus hospitalis* W1, complete genome. DNA, circular, 2137654 bp, [18–21], accession CP002535. Lineage: *Archaea*; Crenarchaeota; Thermoprotei; Sulfolobales; Sulfolobaceae; Acidianus; *Acidianus hospitalis*; *Acidianus hospitalis* W1
**h3**: *Acidilobus saccharovorans* 345-15. complete genome. DNA, circular, 2137654 bp, [18–21], accession CP001742.1. Lineage: *Archaea*; Crenarchaeota; Thermoprotei; Acidilobales; Acidilobaceae; Acidilobus; *Acidilobus saccharovorans*; *Acidilobus saccharovorans* 345-15. Anaerobic bacteria found in hot springs.

to be compared with the following (aerobic/anaerobic) bacteria/fungi:

**b1**: *Mycoplasma putrefaciens* KS1 chromosome, complete genome. DNA, circular, length 832603 bp, [18–21], accession NC 015946,. Lineage: *Bacteria*; Tenericutes; Mollicutes; Mycoplasmatales; Mycoplasmataceae; Mycoplasma; *Mycoplasma putrefaciens*; *Mycoplasma putrefaciens* KS1.
**b2**: *Mortierella verticillata mitochondrion*, complete genome. dsDNA, circular, length 58745 bp, [18–21], accession NC 006838. Lineage: Eukaryota; Opisthokonta; *Fungi*; Fungi incertae sedis; Basal fungal lineages; Mucoromycotina; Mortierellales; Mortierellaceae; Mortierella; *Mortierella verticillata*.
**b3**: *Blattabacterium* sp. (Periplaneta Americana) str. BPLAN, complete genome. DNA, circular, length 636994 nt, [18–21], accession NC 013418. Lineage: *Bacteria*; Bacteroidetes/Chlorobi group; Bacteroidetes; Flavobacteria; Flavobacteriales; Blattabacteriaceae; Blattabacterium; Blattabacterium sp. (Periplaneta Americana); *Blattabacterium* sp. (Periplaneta Americana) str. BPLAN.

Moreover we will compare DNA sequences with artificial sequences of nucleotides randomly taken (see Section 4).

### 2.1. Archaea

Archaea are a group of elementary single-cell microorganisms, having no cell nucleus or any other membrane-bound organelles within their cells. They are similar to bacteria, since they have the same size and shape (apart few exceptions) and the generally similar cell structure. However, the evolutionary history of archaea and their biochemistry has significant differences with regard to other forms of life. Therefore they are considered as members of a phylogenetic group distinct from bacteria and eukaryota.

Archaea during their evolution have been spreading all over the Earth in almost all habitats [22, 23] existing in a broad range of habitats, being one of the major contribution (20%) to earth's biomass. The most peculiar feature of archaea is that they can live in some environments with extreme life conditions (thus being considered as extremophiles [22, 24]). Indeed, some archaea survive to high temperatures, over 100°C, while others can live in very cold habitats or highly saline, acidic, or alkaline water. Nevertheless some archaea are living in mild conditions.

It has been also recognized that the archaea may be the most ancient organisms on the Earth, so that archaea, and eukaryotes are probably diverged early from an ancestral colony of organisms.

We will see, in the following, that archaea DNA it looks very close to random sequences so that we can assume that the ancestral organism were evolving by random permutations from a primitive assembly of nucleotides. So that the evolution can be seen as a tendency to a steady state far from the randomness. Therefore, the bacteria's DNA (and other eukaryotes' [1–6]), as a result of the evolution, shows the existence of some hidden stability.

## 3. Correlation Plots

In this section we will consider some elementary plots from where it is possible to visualize autocorrelation, distribution law of nucleotides and to measure some fundamental parameters by using frequency count.

Let

(1) $$\begin{array}{c} 𝒜\overset{\mathrm{def}}{=}\left\{\text{A},\text{C},\text{G},\text{T}\right\} \end{array}$$

be the finite set (alphabet) of nucleotides (nucleic acids): adenine (A), cytosine (C), guanine (G), thymine (T), and *x* ∈ *𝒜* any member of the alphabet. Nucleic acids are further grouped according to their ligand properties as

- purine {A, G}, pyrimidine {C, T},
- amino {A, C}, keto {G, T},
- weak hydrogen bonds {A, T}, strong hydrogen bond {G, C}.

A DNA sequence is the finite symbolic sequence

(2) $$\begin{array}{c} 𝒮=\mathbb{N}\times 𝒜 \end{array}$$

so that

(3) $$\begin{array}{c} 𝒮≝{\left\{x_{h}\right\}}_{h=1,\ldots ,N},\;N<\infty \end{array}$$

with

(4) $$\begin{array}{c} x_{h}≝\left(h,x\right)=x\left(h\right),\;\left(h=1,2,\ldots ,N;\;x\in 𝒜\right) \end{array}$$

being the nucleotide *x* at the position *h*.

In general we can define an *ℓ*-length alphabet as follows: let the *ℓ*-length DNA word be defined by the *ℓ*-combination of the 4 nucleotides (1). For each fixed length *ℓ* there are 4^*ℓ*^ words, however not all of them can be considered, from biological point of view, as independent instances (see, e.g., Table 1), for this we define the *ℓ*-length alphabet as the set of *ℓ*-length independent words:

(5) $$\begin{array}{c} {𝒜}_{\ell }≝\left\{a_{1},a_{2},\ldots ,a_{M_{\ell }}\right\},\;\;M_{\ell }≝\left|{𝒜}_{\ell }\right|\leq 4^{\ell } \end{array}$$

with |⋯| cardinality of the set and

(6) $$\begin{array}{c} \ell ≝\text{length}\left(a_{j}\right),\;\left(j=1,\ldots ,M_{\ell }\right). \end{array}$$

For instance with *ℓ* = 1, the alphabet is *𝒜*_1_ = *𝒜* = {A, C, G, T}, with *ℓ* = 3 the alphabet is given by the 20 amino acids

(7) $$\begin{array}{c} {𝒜}_{3}=\left\{\text{M},\text{E},\text{Q},\text{D},\text{R},\text{T},\text{N},\text{H},\text{V},\text{G},\text{L},\text{S},\text{P},\text{F},\text{I},\text{C},\text{A},\text{K},\text{Y},\text{W}\right\} \end{array}$$

each amino acid being represented by a 3-length word of Table 1.

Let *𝒮*_*N*_ be an *N*-length ordered sequence of nucleotides {A, C, G, T} and *𝒜*_*ℓ*_ the chosen alphabet, a DNA sequence of words is the finite symbolic sequence

(8) $$\begin{array}{c} {𝒟}_{\ell }\left(S_{N}\right)=\mathbb{N}\times {𝒜}_{\ell } \end{array}$$

so that

(9) $$\begin{array}{c} {𝒟}_{\ell }\left(S_{N}\right)\overset{\mathrm{def}}{=}{\left\{x_{h}\right\}}_{h=1,\ldots ,N},\;\left(x\in {𝒜}_{\ell };\;N<\infty \right) \end{array}$$

with

(10) $$\begin{array}{c} x_{h}\overset{\mathrm{def}}{=}\left(h,x\right),\;\left(h=1,2,\ldots ,N;\;x\in {𝒜}_{\ell }\right) \end{array}$$

being the word *x* at the position *h*.

### 3.1. Indicator Matrix

The 2D indicator function, based on the 1D definition given in [25], is the map

(11) $$\begin{array}{c} u:𝒮\times 𝒮\to \left\{0,1\right\} \end{array}$$

such that

(12) $$\begin{array}{c} u\left(x_{h},x_{k}\right)\overset{\mathrm{def}}{=}\{\begin{array}{cc} 1 & \text{if}\;x_{h}=x_{k},\\ 0 & \text{if}\;x_{h}\neq x_{k}, \end{array}\;\left(x_{h}\in 𝒮,\;x_{k}\in 𝒮\right), \end{array}$$

with

(13) $$\begin{array}{c} u\left(x_{h},x_{k}\right)=u\left(x_{k},x_{h}\right),\;\;u\left(x_{h},x_{h}\right)=1 \end{array}$$

and, where for short, we have assumed

(14) $$\begin{array}{c} 𝒮\overset{\mathrm{def}}{=}{𝒟}_{1}\left(S_{N}\right). \end{array}$$

According to (12), the indicator of an *N*-length sequence can be easily represented by the *N* × *N* sparse symmetric matrix of binary values {0,1} which results from the indicator matrix (see also [3–5])

(15) $$\begin{array}{c} u_{hk}\overset{\mathrm{def}}{=}u\left(x_{h},x_{k}\right),\;\left(x_{h}\in 𝒮,\;x_{k}\in 𝒮;\;h,k=1,\ldots ,N\right), \end{array}$$

being, explicitly

(16) $$\begin{array}{c} \begin{array}{cccccccccccc} \vdots & \vdots & \vdots & \vdots & \vdots & \vdots & \vdots & \vdots & \vdots & \vdots & \vdots & ⋰\\ \text{G} & 0 & 1 & 0 & 0 & 0 & 0 & 0 & 0 & 0 & 1 & \cdots \\ \text{C} & 0 & 0 & 0 & 1 & 0 & 0 & 0 & 0 & 1 & 0 & \cdots \\ \text{A} & 1 & 0 & 0 & 0 & 1 & 0 & 1 & 1 & 0 & 0 & \cdots \\ \text{A} & 1 & 0 & 0 & 0 & 1 & 0 & 1 & 1 & 0 & 0 & \cdots \\ \text{T} & 0 & 0 & 1 & 0 & 0 & 1 & 0 & 0 & 0 & 0 & \cdots \\ \text{A} & 0 & 0 & 0 & 0 & 1 & 0 & 0 & 1 & 0 & 0 & \cdots \\ \text{C} & 0 & 0 & 0 & 1 & 0 & 0 & 0 & 0 & 1 & 0 & \cdots \\ \text{T} & 0 & 0 & 1 & 0 & 0 & 1 & 0 & 0 & 0 & 0 & \cdots \\ \text{G} & 0 & 1 & 0 & 0 & 0 & 0 & 0 & 0 & 0 & 1 & \cdots \\ \text{A} & 1 & 0 & 0 & 0 & 1 & 0 & 0 & 1 & 0 & 0 & \cdots \\ u_{hk} & \text{A} & \text{G} & \text{T} & \text{C} & \text{A} & \text{T} & \text{A} & \text{A} & \text{C} & \text{G} & \cdots \end{array} \end{array}$$

**Figure 1:**
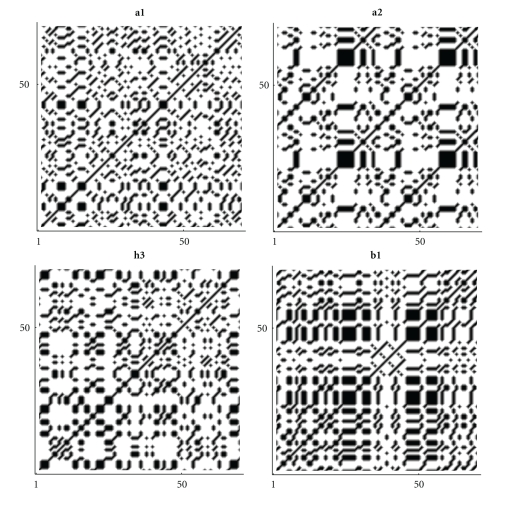
Indicator matrix for: (**a1**) pseudorandom 70-length sequence; (**a2**) pseudo-periodic 70-length sequence with period *π* = 35; (**b1**) 70-length DNA sequence of Mycoplasma KS1 bacter; (**h3**) 70-length DNA sequence of Acidilobus Archaea.

This squared matrix can be plotted in 2 dimensions by putting a black dot where *u*_*hk*_ = 1 and white spot when *u*_*hk*_ = 0 (Figure 1) thus giving rise to the two-dimensional dot plot, which is a special case of the *recurrence plot* [26].

A simple generalization of this matrix can be considered for the alphabets *𝒜*_*ℓ*_, as follows. By choosing the 3 alphabet of amino acids, the 2D indicator function is the map

(17) $$\begin{array}{c} u:{𝒟}_{3}\left(S_{N}\right)\times {𝒟}_{3}\left(S_{N}\right)\to \left\{0,1\right\} \end{array}$$

such that

(18) $$\begin{array}{c} u\left(x_{h},x_{k}\right)\overset{\mathrm{def}}{=}\{\begin{array}{cc} 1 & \text{if}\;x_{h}=x_{k},\\ 0 & \text{if}\;x_{h}\neq x_{k}, \end{array}\;\left(x_{h}\in {𝒟}_{3}\left(S_{N}\right),\;x_{k}\in {𝒟}_{3}\left(S_{N}\right)\right), \end{array}$$

with

(19) $$\begin{array}{c} u\left(x_{h},x_{k}\right)=u\left(x_{k},x_{h}\right),\;\;u\left(x_{h},x_{h}\right)=1. \end{array}$$

According to (12), the indicator, on the 3-alphabet of amino acids of an *N*-length sequence can be easily represented by the *N* × *N* sparse symmetric matrix of binary values {0,1}:

(20) $$\begin{array}{cc} & u_{hk}\overset{\mathrm{def}}{=}u\left(x_{h},x_{k}\right),\\ & \left(x_{h}\in {𝒟}_{3}\left(S_{N}\right),\;x_{k}\in {𝒟}_{3}\left(S_{N}\right);\;h,k=1,\ldots ,N\right), \end{array}$$

being, explicitly

(21) $$\begin{array}{c} \begin{array}{cccccccccccc} \vdots & \vdots & \vdots & \vdots & \vdots & \vdots & \vdots & \vdots & \vdots & \vdots & \vdots & ⋰\\ \text{M} & 1 & 0 & 0 & 0 & 0 & 0 & 0 & 0 & 0 & 1 & \cdots \\ \text{Q} & 0 & 0 & 0 & 0 & 0 & 0 & 0 & 0 & 1 & 0 & \cdots \\ \text{R} & 0 & 0 & 1 & 1 & 0 & 0 & 0 & 1 & 0 & 0 & \cdots \\ \text{T} & 0 & 0 & 0 & 0 & 0 & 1 & 1 & 0 & 0 & 0 & \cdots \\ \text{T} & 0 & 0 & 0 & 0 & 0 & 1 & 1 & 0 & 0 & 0 & \cdots \\ \text{E} & 0 & 0 & 0 & 0 & 1 & 0 & 0 & 1 & 0 & 0 & \cdots \\ \text{R} & 0 & 0 & 1 & 1 & 0 & 0 & 0 & 0 & 0 & 0 & \cdots \\ \text{R} & 0 & 0 & 1 & 1 & 0 & 0 & 0 & 1 & 0 & 0 & \cdots \\ \text{K} & 0 & 1 & 0 & 0 & 0 & 0 & 0 & 0 & 0 & 0 & \cdots \\ \text{M} & 1 & 0 & 0 & 0 & 0 & 0 & 0 & 0 & 0 & 1 & \cdots \\ u_{hk} & \text{M} & \text{K} & \text{R} & \text{R} & \text{E} & \text{T} & \text{T} & \text{R} & \text{Q} & \text{M} & \cdots \end{array} \end{array}$$

With the graphical representation of this matrix we can also show the correlation of amino acids.

### 3.2. Test Sequences

In the following, in order to single out the main features of biological sequences, we will compare the DNA sequence with some test sequences.

1. Pseudorandom *N*-length sequence of nucleotides is the sequence {*ℛ*_*i*_}_*i*=1,…,*N*_^*ℓ*^ where *r*_*i*_ is a symbol randomly chosen in the alphabet *𝒜*_*ℓ*_, like for example, (*ℓ* = 1):
  (22) $$\begin{array}{c} \left\{\text{A},\text{C},\text{A},\text{G},\text{T},\text{A},\text{T},\text{G},\text{G},\text{A},\text{T},\text{T},\text{A},\text{C},\text{C},\text{G},\ldots \right\} \end{array}.$$
2. Pseudoperiodic *N*-sequence of nucleotides with period *π* is the direct sum of a given *π*-length pseudorandom sequence, such that *N* = *kπ*, (*k* ∈ *ℕ*) and *ℛ*_*i*_ = *ℛ*_*i*+*π*_, for example,
  (23) $$\begin{array}{cc} & \left\{\text{A},\text{C},\text{A},\text{G},\text{A},\text{C},\text{A},\text{G},\text{A},\text{C},\text{A},\text{G},\text{A},\text{C},\text{A},\text{G},\ldots \right\},\\ & \;\;\;\;\;\;\;\;\;\;\;\;\left(\pi =4\right). \end{array}$$
  When *π* = 1 we have a pseudorandom sequence.

If we plot the indicator matrix of some bacteria and compare it with a pseudorandom and periodic sequence, we can see that (Figure 1)

1. the main diagonal is a symmetry axis for the plot;
2. there are some motifs which are repeated at different scales like in a fractal;
3. periodicity is detected by parallel lines to the main diagonal (Figure 1(**a2**));
4. empty spaces are more distributed than filled spaces, in the sense that the matrix *u*_*hk*_ is a sparse matrix (having more 0's than 1's);
5. it seems that there are some square-like islands where black spots are more concentrated; these islands show the persistence of a nucleotide (Figures 1(**a2**) and 1(**b1**));
6. the dot plot of archaea is very similar to the dot plot of a random sequence (Figures 1(**a1**) and 1(**h3**)).

It can be noticed that DNA sequences of a living organism resemble (Figure 1) random sequences, with some short range influence, built on the same alphabet. This has been taken as an axiom of nucleotides distribution, so that DNA sequences are often considered as Markov chain [27]. However, there are some hidden rules in combining the nucleotides and these rules lead, during the evolution, to a steady distribution. In fact, the more primitive the sequence is, the more randomly distributed the nucleotides are. It seems that as a consequence of the evolution, nucleotides move from a disordered aggregation toward a more organized structure, shown by the growing islands in the dot plot. The biological evolution is such that the challenge for the self-organization might follow from random permutations of a primitive disordered sequence so that the organization, that is, the complexity, is only the result of many arbitrary permutations of randomness. During the challenge for complexity, DNA sequence becomes “less random” and it loses some kind of energy.

**Figure 2:**
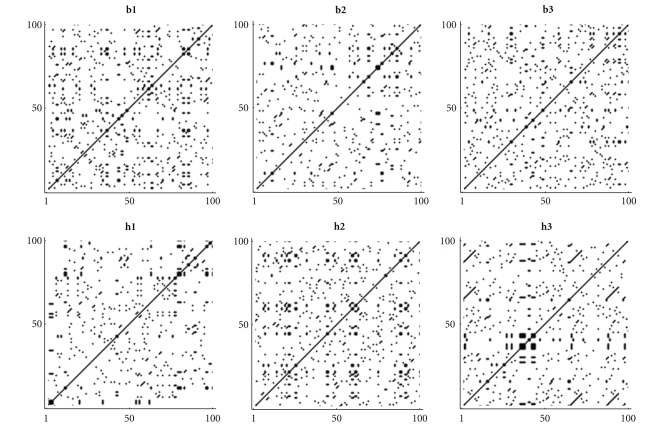
Indicator matrix for the first 100 amino acids of (**h1**) *Aeropyrum pernix* K1, (**h2**) *Acidianus hospitalis* W1, (**h3**) *Acidilobus saccharovorans* 345-15 (**b1**) *Mycoplasma putrefaciens* KS1, (**b2**) *Mortierella verticillata*, and (**b3**) *Blattabacterium* sp.

From the graphical representation of the indicator matrix for bacteria and amino acids we can see a more sparse matrix, but with some typical plots (Figure 2).

### 3.3. Spiral Plot

In this section we consider a 2D distribution of nucleotides, following the idea given by Ulam for the distribution of primes, along an Ulam-like spiral [28]. In order to find some patterns in their distribution, nucleotides are arranged along a rectangular spiral. This is equivalent to mapping the 1D sequence of integers into a 2D sequence as follows:

(24) $$\begin{array}{ccc} X_{1} & \;\;1 & \left\{0,0\right\}\\ X_{2} & \;\;2 & \left\{1,0\right\}\\ X_{3} & \;\;3 & \left\{1,1\right\}\\ X_{4} & \;\;4 & \left\{0,1\right\}\\ X_{5} & \;\;5 & \left\{-1,1\right\}\\ X_{6} & \;\;6 & \left\{-1,0\right\}\\ X_{7} & \;\;7 & \left\{-1,-1\right\}\\ X_{8} & \;\;8 & \left\{0,-1\right\}\\ X_{9} & \;\;9 & \left\{1,-1\right\}\\ X_{10} & \;\;10 & \left\{2,-1\right\}\\ X_{11} & \;\;11 & \left\{2,0\right\}\\ \vdots & \;\;\vdots & \vdots \end{array}$$

**Figure 3:**
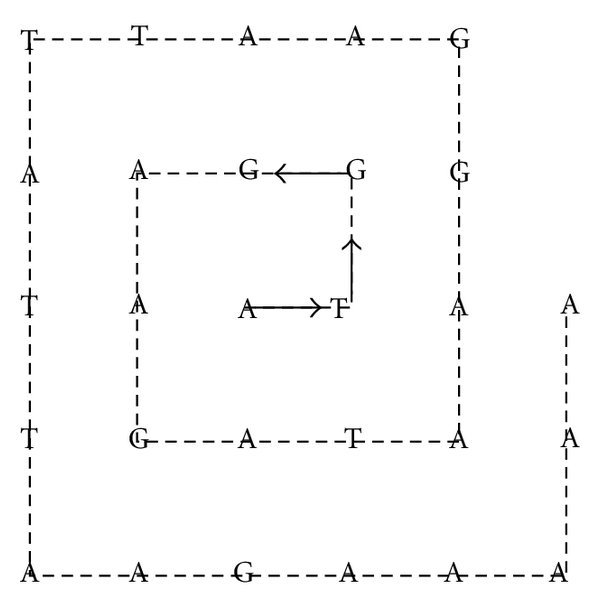
Distribution of nucleotides on a rectangular spiral.

For instance the sequence

(25) $$\begin{array}{c} \left\{\text{A},\text{T},\text{G},\text{G},\text{A},\text{A},\text{G},\text{A},\text{T},\text{A},\text{A},\text{G},\ldots \right\} \end{array}$$

distributed along the spiral looks like Figure 3.

For each nucleotide we can draw a spiral containing the distribution of only one acid nucleic. To each organism there correspond four plots, for A, C, G, T, respectively.

**Figure 4:**
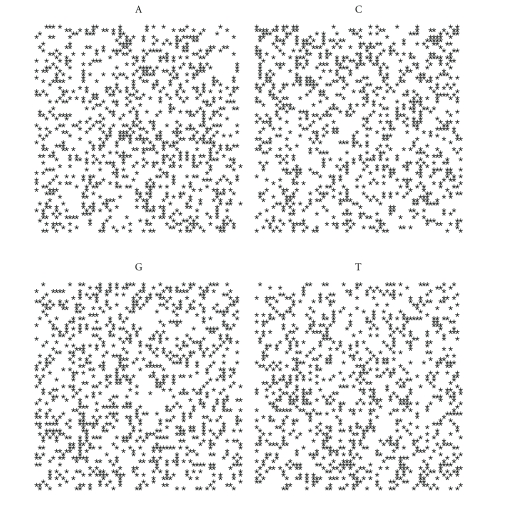
Spiral distribution of the first 3752 nucleotides for the random sequence.

Let us first note that on a random sequence (Figure 4) the four distribution are equivalent.

**Figure 5:**
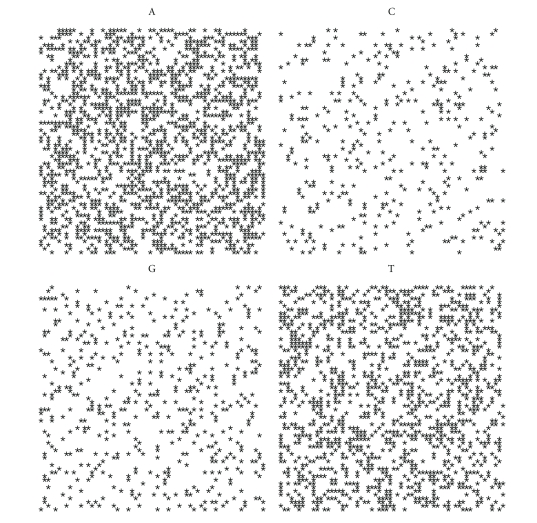
Spiral distribution of the first 3752 nucleotides for *Mycoplasma putrefaciens* KS1.

**Figure 6:**
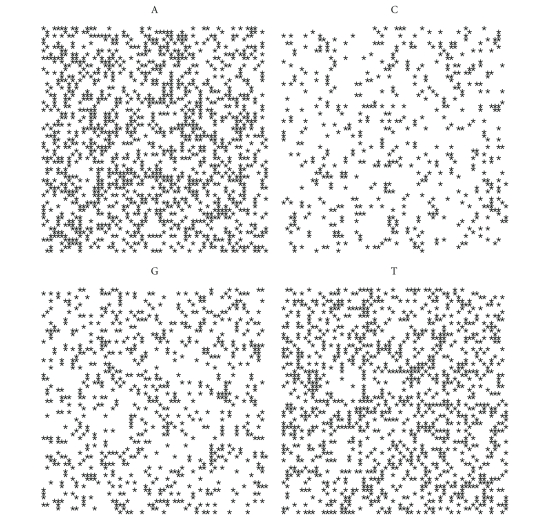
Spiral distribution of the first 3752 nucleotides for *Mortierella verticillata*.

**Figure 7:**
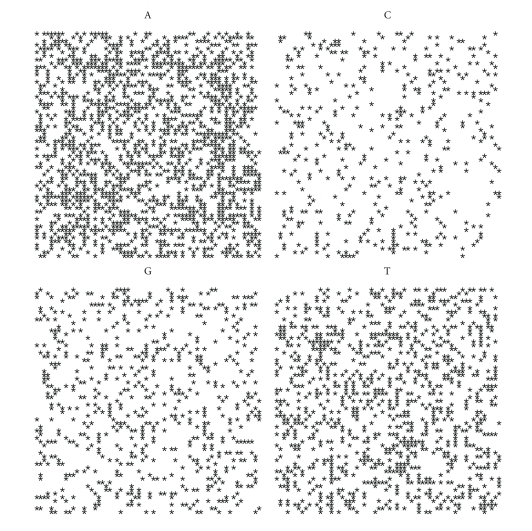
Spiral distribution of the first 3752 nucleotides for *Blattabacterium* sp.

**Figure 8:**
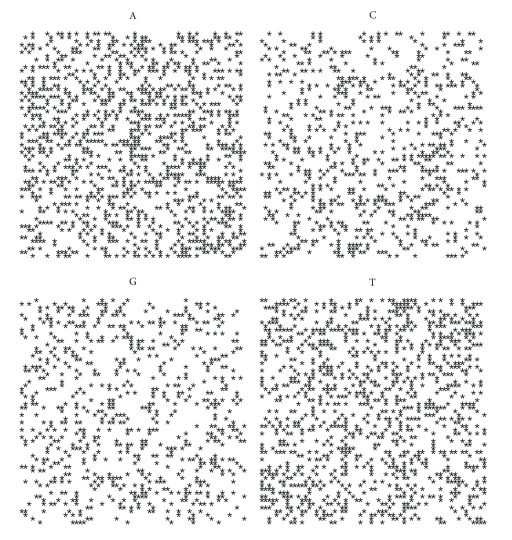
Spiral distribution of the first 3752 nucleotides for *Aeropyrum pernix* K1..

**Figure 9:**
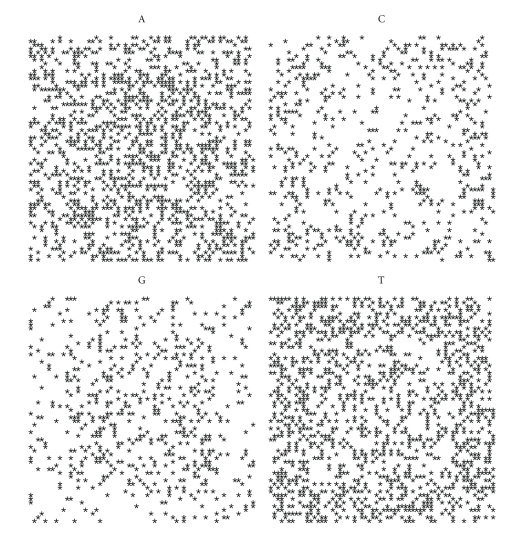
Spiral distribution of the first 3752 nucleotides for *Acidianus hospitalis* W1.

**Figure 10:**
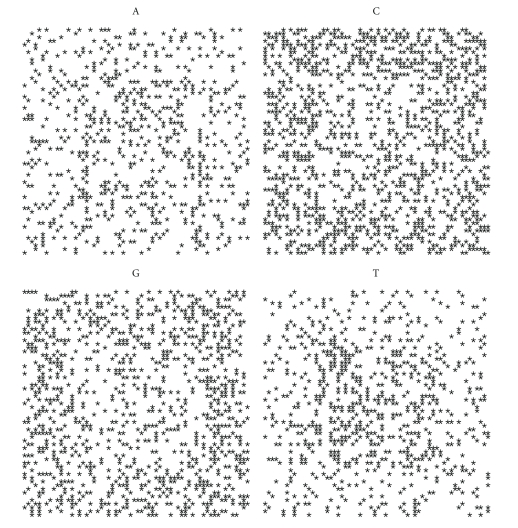
Spiral distribution of the first 3752 nucleotides for *Acidilobus saccharovorans* 345-15.

By comparing the spirals of bacteria, random and archaea (Figures 4, 5, 6, 7, 8, 9, 10) we can see that there is a different distribution of each nucleotide. However the more evolved organism tends to have a higher percentage of weak hydrogen bonds (Figures 5, 6 and 7), so that we can assume the following.

**Conjecture 1**

During the evolution, the distribution of nucleotides changes in a such way that strong hydrogen bonds tend to become weak.

It should be noticed that along these spirals, there is a one-to-one map *λ* between *ℕ* and the points of the spiral (with integer coordinates) in *ℜ* ^2^

(26) $$\begin{array}{c} \lambda :\mathbb{N}\mapsto \gamma \subset ℜ\times ℜ \end{array}$$

so that

(27) $$\begin{array}{cc} & \lambda \left(n\right)=\left(a,b\right),\;\left(n\in \mathbb{N};\;\left(a,b\right)\in \gamma \subset ℜ\times ℜ;\;a\in \mathbb{Z},\;b\in \mathbb{Z}\right),\\ & \lambda^{-1}\left(a,b\right)=n. \end{array}$$

This bijective map can be considered also between *ℕ* and the complex space *ℂ* so that each natural number corresponds to a complex number (with integer coefficients)

(28) $$\begin{array}{c} \lambda \left(n\right)=z\overset{\mathrm{def}}{=}a+ib,\;\left(n\in \mathbb{N};\;a,b\in \mathbb{Z};\;z\in \mathbb{C}\right). \end{array}$$

Since these spirals seem to fill in a finite region of the plane we can evaluate the complexity of each curve by typical fractal measures.

## 4. Parameters of Complexity

In this section we define some parameters, based on frequency distribution, which can measure the complexity of a DNA by computing the complexity of its representation in the complex plane (for a more detailed analysis see [29] and references therein).

Let *𝒮*_*N*_ be an *N*-length-ordered sequence of nucleotides, and

(29) $$\begin{array}{c} p_{x}\left(h\right),\;x\in {𝒜}_{1}=\left\{\text{A},\text{C},\text{G},\text{T}\right\} \end{array}$$

be the probability to find the nucleotide *x* at the position *h*, 1 ≤ *h* ≤ *N*. According to (12) we define

(30) $$\begin{array}{c} \begin{array}{cc} a_{h}\overset{\mathrm{def}}{=}\overset{h}{\underset{j=1}{\sum }}u_{Aj}, & c_{h}\overset{\mathrm{def}}{=}\overset{h}{\underset{j=1}{\sum }}u_{Cj},\\ g_{h}\overset{\mathrm{def}}{=}\overset{h}{\underset{j=1}{\sum }}u_{Gj}, & t_{h}\overset{\mathrm{def}}{=}\overset{h}{\underset{j=1}{\sum }}u_{Tj},\\ & \;\;\;\;\left(1\leq h\leq N\right) \end{array} \end{array}$$

as the number of nucleotides in the *h*-length segment of *𝒮*_*N*_, so that

(31) $$\begin{array}{c} a_{h}+c_{h}+g_{h}+t_{h}=h \end{array}.$$

The corresponding frequencies are

(32) $$\begin{array}{c} v_{x}\left(h\right)\overset{\mathrm{def}}{=}\frac{1}{h}\overset{h}{\underset{j=1}{\sum }}u_{xj},\;x\in A_{1},\;\left(1\leq h\leq N\right), \end{array}$$

so that

(33) $$\begin{array}{c} v_{A}\left(h\right)=\frac{a_{h}}{h},\;\;v_{C}\left(h\right)=\frac{c_{h}}{h},\\ v_{G}\left(h\right)=\frac{g_{h}}{h},\;\;v_{T}\left(h\right)=\frac{t_{h}}{h}. \end{array}$$

We can assume that for large sequences

(34) $$\begin{array}{c} p_{x}\left(h\right)≅v_{x}\left(h\right) \end{array}.$$

### 4.1. Randomness

Since for a random sequence the frequencies of nucleotides coincide for large *n*,

(35) $$\begin{array}{c} v_{A}\left(n\right)≅v_{C}\left(n\right)≅v_{G}\left(n\right)≅v_{T}\left(n\right) \end{array}$$

we can define as randomness index the following:

(36) $$\begin{array}{c} ℛ\overset{\mathrm{def}}{=}1-\sigma \left(v_{A}\left(n\right),v_{C}\left(n\right),v_{G}\left(n\right),v_{T}\left(n\right)\right) \end{array}$$

with *σ* being the variance, so that *ℛ* = 1 for random sequence and *ℛ* = 0 for a nonrandom sequence. Over the first 10000 nucleotides we have the randomness value of Table 2.

However, if we compute the randomness index over the frequencies of amino acids in the *𝒜*_3_ alphabet then we can observe a different distribution of values. Over the first 30000 nucleotides corresponding to 10000 amino acids, we have the randomness value of Table 3.

So that we can comment that the arising complexity of the words and alphabets shows a different randomness in each alphabet.

### 4.2. Complexity

As a simple measure of complexity [30–32], for an *n*-length sequence, the following has been proposed [33]:

(37) $$\begin{array}{c} K=\frac{1}{n}\log\frac{n!}{a_{n}!c_{n}!g_{n}!t_{n}!}. \end{array}$$

In Table 4 the complexity of the first 100-length segment of the DNA sequences is computed. It is interesting to notice the more similarities between the archaea Acidilobus with the pseudorandom sequence than with the pseudoperiodic. Nucleotide distribution in primitive biosequences is more likely random than pseudodeterministic. Moreover, the evolution reduces the complexity of the sequence.

### 4.3. Fractal Dimension

The fractal dimension is computed on the dot plot, by the box counting algorithm [34, 35], as the average of the number *p*(*n*) of 1's in the randomly taken *n* × *n* minors of the *N* × *N* indicator matrix *u*_*hk*_ or equivalently the number *p*(*n*) of black dots in the randomly taken *n* × *n* squares over the dot plot

(38) $$D=\frac{1}{2N}\overset{N}{\underset{n=2}{\sum }}\;\frac{\log p\left(n\right)}{\log n}.$$

The explicit computation enables us to compare the fractal dimension on the first 100-length segments of DNA chains, with an approximation up to 10^−3^ (see Table 5).

If we compare the fractal dimensions of the bacteria with pseudorandom and pseudoperiodic we can see that the fractal dimension of nucleotide distribution ranges, for all variants, in the interval [1.28–1.30]. As expected, the more “random” sequences have higher fractal dimension.

### 4.4. Entropy

Another fundamental parameter, related to the information content of a sequence which measures the heterogeneity of data, is the information entropy (or Shannon entropy) [36–42]. Based on the axiom that less information implies a larger uncertainty and vice versa that more information leads us to a more deterministic model, the entropy concept has been recently offering some interesting interpretations about uncertainty in DNA. In fact, DNA as any other signal has been considered as a sequence of symbols carrying chemical-functional information.

The normalized Shannon entropy [39, 40, 42] is defined, over the alphabet *𝒜*_*ℓ*_, as

(39) $$H\left(n\right)=-\frac{1}{\log\ell }\underset{x\in {𝒜}_{\ell }}{\sum }p_{x}\left(n\right)\times \{\begin{array}{cc} \log p_{x}\left(n\right) & \text{if}\;p_{x}\left(n\right)\neq 0,\\ 0 & \text{if}\;p_{x}\left(n\right)=0, \end{array}$$

where *p*_*x*_(*n*) should be computed for large sequences. According to (32), (34), we will approximate its value with

(40) $$p_{x}\left(n\right)≅\frac{1}{n}\overset{n}{\underset{i=1}{\sum }}u_{xi},\;\left(x\in {𝒜}_{\ell },\;1\leq n\leq N\right).$$

However, the entropy is a parameter very similar to the complexity. In fact, it can be easily seen that (for the proof see [29]) the entropy *H* and the measure of complexity *K* differ for a factor. There follows that the entropy does not give any new information comparing with the previous parameters. As expected also the table of entropies classifies bacteria and archaea in the same way (Table 6).

## 5. Complex Root Representation of DNA Words

The complex (digital) representation of a DNA sequence of words is the map of the symbolic sequence of words into a set of complex numbers and it is defined as

(41) $$\begin{array}{c} {𝒟}_{\ell }\left(S_{N}\right)\overset{\rho }{\to }\mathbb{C} \end{array}$$

such that for each *x*_*h*_ ∈ *𝒟*_*ℓ*_(*S*_*N*_) it is *ρ*(*x*_*h*_) ∈ *ℂ*.

The complex root representation of the sequence *S*_*N*_ is the sequence *𝒟*_*ℓ*_(*S*_*N*_) of complex numbers {*y*_*h*_}_*h*=1,…,*N*_ defined as

(42) $$\begin{array}{c} y_{h}=\rho \left(x_{h}\right)\overset{\mathrm{def}}{=}e^{2\pi i(j-1)/|{𝒜}_{\ell }|},\;\left(j=1,\ldots ,\left|{𝒜}_{\ell }\right|,\;h=1,\ldots ,N\right) \end{array}$$

with $i=\sqrt{-1}$ being the imaginary unit. There follows that, independently on the alphabet, it is

(43) $$\begin{array}{c} |y_{h}|=\left|e^{2\pi i(j-1)/|{𝒜}_{\ell }|}\right|=1,\;\left(\forall \ell ;\;h=1,\ldots ,N\right)\; \end{array}$$

being all complex roots, of the unit, located on the unit circle of the complex plane *ℂ* ^1^.

For instance, with *𝒜*_1_ = {A, C, G, T}, the cardinality of the alphabet is |*𝒜*_1_ | = 4 and

(44) $$\begin{array}{c} \rho \left(\text{A}\right)=e^{0/4}=1,\;j=1,\\ \rho \left(\text{C}\right)=e^{\pi i/2}=i,\;j=2,\\ \rho \left(\text{G}\right)=e^{\pi i}=-1,\;j=3,\\ \rho \left(\text{T}\right)=e^{\pi i3/2}=-i,\;j=4. \end{array}$$

Analogously, with *𝒜*_3_ = {*M*, *E*,…, *W*} it is |*𝒜*_3_ | = 20 and the 20 complex roots of unit

(45) $$\begin{array}{c} \rho \left(x_{n}\right)=e^{2\pi i(n-1)/20},\;\left(n=1,\ldots ,20;\;x_{n}\in {𝒜}_{3}\right) \end{array}$$

so that explicitly is

(46) $$\begin{array}{ccc} & \rho \left(M\right)=e^{2\pi i0/20}=1, & j=1,\\ & \rho \left(E\right)=e^{\pi i/10}=\frac{1}{4}\left[\sqrt{2\left(5+\sqrt{5}\right)}+i\left(\sqrt{5}-1\right)\right], & j=2,\\ & \rho \left(Q\right)=e^{\pi i/5}=\frac{1}{4}\left[1+\sqrt{5}+i\sqrt{2\left(5-\sqrt{5}\right)}\right], & j=3,\\ & \;\;\vdots & \vdots \;\\ & \rho \left(W\right)=e^{\pi i19/10}=\frac{1}{4}\left[\sqrt{2\left(5+\sqrt{5}\right)}-i\left(\sqrt{5}-1\right)\right], & j=20. \end{array}$$

Therefore the complex representation of a DNA sequence is a sequence of complex numbers

(47) $$\begin{array}{c} y_{h}=\xi_{h}+\eta_{h}i,\;\xi_{h}=ℜ\left(y_{h}\right),\;\eta_{h}=ℑ\left(y_{h}\right) \end{array}$$

with *y*_*h*_ given by (42).

An *n*-length pseudorandom (white noise) complex sequence belonging to the unit circle can be defined directly by using some random exponents

(48) $$\begin{array}{c} R_{n}\overset{\mathrm{def}}{=}{\left(-1\right)}^{r_{n}}i^{s_{n}},\;\left|R_{n}\right|=1, \end{array}$$

with *r*_*n*_, *s*_*n*_ being random values in the set {0, *ℕ*}.

### 5.1. Random Walks

Random walk on the complex sequence **Y**_*N*_ is defined as the series **Z**_*N*_ = {*z*_*n*_}_*n*=1,…,*N*_

(49) $$z_{n}\overset{\mathrm{def}}{=}\underset{k=1,\ldots ,n}{\sum }y_{k},\;n=1,\ldots ,N$$

which is the cumulative sum

(50) $$\left\{y_{1},y_{1}+y_{2},\;\ldots ,\overset{n}{\underset{s=1}{\sum }}y_{s}\ldots ,\overset{N}{\underset{s=1}{\sum }}y_{s}\right\}.$$

When *y*_*k*_ = *ρ*(*x*_*k*_) with *x*_*k*_ ∈ *𝒜*_*ℓ*_ and **X**_*k*_ ∈ *S*_*N*_ we will properly call these walks as DNA walk. When the *y*_*k*_ are randomly generated we will call them random walks.

By remembering the definition of frequencies, DNA walk is the complex value signal {*Z*_*n*_}_*n*=0,…,*N*−1_ with

(51) $$z_{n}=\left(ℜ\left[z_{n}\right],ℑ\left[z_{n}\right]\right)=\left(a_{n}-g_{n}\right)+\left(t_{n}-c_{n}\right)i,\;z_{n}\in {\mathbb{C}}_{1},$$

where the coefficients *a*_*n*_, *g*_*n*_, *t*_*n*_, *c*_*n*_ given by (12) fulfill the condition (31).

**Figure 11:**
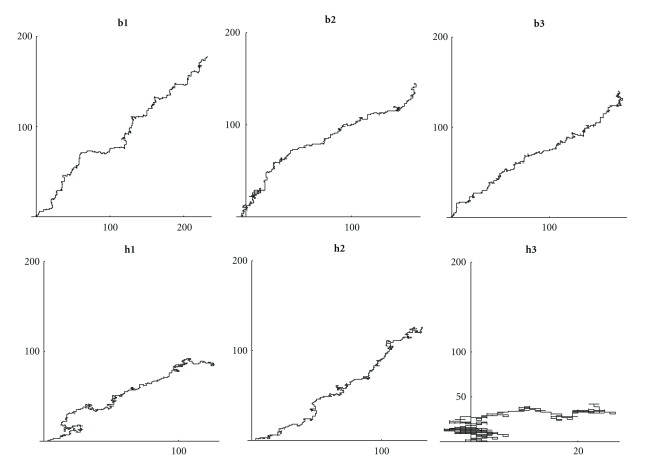
Walks on the first 200 nucleotides: (**b1**) *Mycoplasma putrefaciens*, (**b2**) *Mortierella verticillata*, (**b3**) *Blattabacterium*, (**h1**) *Aeropyrum pernix*, (**h2**) *Acidianus hospitalis*, and (**h3**) *Acidilobus saccharovorans*.

If we compare the DNA walks (Figure 11) some primitive archaea such as h3 are very similar to a random walk (Figure 13). In particular archaea seem to grow less than other bacteria (with the exception of b2).

**Figure 12:**
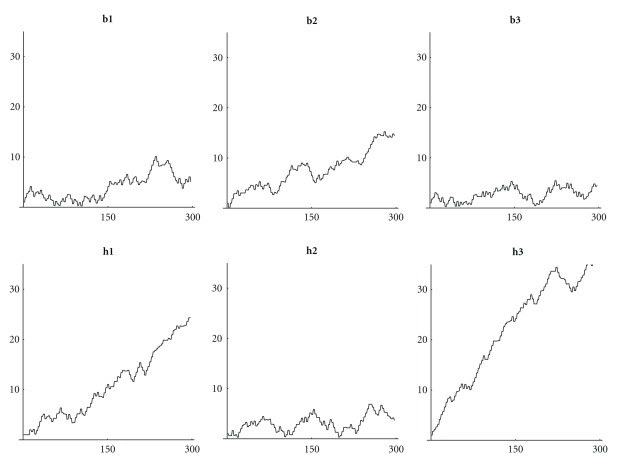
Absolute value of walks on the first 100 amino acids: (**b1**) *Mycoplasma putrefaciens*, (**b2**) *Mortierella verticillata*, (**b3**) *Blattabacterium*, (**h1**) *Aeropyrum pernix*, (**h2**) *Acidianus hospitalis*, (**h3**) *Acidilobus saccharovorans*.

It is interesting also to notice that the random walks on amino acids (Figure 12) show that more evolved organisms have some “periodic” behavior, while the absolute value of walks on archaea is growing fast.

## 6. Wavelet Analysis

Wavelet analysis is a powerful method extensively applied to the analysis of biological signals [12, 43–45] aiming to single out the most significant parameters of complexity and heterogeneity in a time series and, in particular, in a DNA sequence. This method is based on the analysis of wavelet coefficients which are obtained by the wavelet transform.

We will consider in the following the Haar wavelet basis (see, e.g., [3, 4, 29]) made by scaling functions:

(52) $$\begin{array}{c} \begin{array}{c} \varphi_{k}^{n}\left(x\right)\overset{\mathrm{def}}{=}2^{n/2}\varphi \left(2^{n}x-k\right),\;\left(0\leq n,\;0\leq k\leq 2^{n}-1\right),\\ \varphi \left(2^{n}x-k\right)=\{\begin{array}{cc} 1, & x\in \Omega_{k}^{n},\;\Omega_{k}^{n}\overset{\mathrm{def}}{=}\left[\frac{k}{2^{n}},\frac{k+1}{2^{n}}\right),\\ 0, & x\notin \Omega_{k}^{n}, \end{array} \end{array} \end{array}$$

and the *Haar wavelets*:

(53) $$\begin{array}{c} \begin{array}{c} \psi_{k}^{n}\left(x\right)\overset{\mathrm{def}}{=}2^{n/2}\psi \left(2^{n}x-k\right),\;{||\psi_{k}^{n}\left(x\right)||}_{L^{2}}=1,\\ \psi \left(2^{n}x-k\right)\overset{\mathrm{def}}{=}\{\begin{array}{cc} -1, & x\in \left[\frac{k}{2^{n}},\frac{k+1/2}{2^{n}}\right),\\ 1, & x\in \left[\frac{k+1/2}{2^{n}},\frac{k+1}{2^{n}}\right),\\ & \left(0\leq n,\;0\leq k\leq 2^{n}-1\right),\\ 0, & \text{elsewhere}. \end{array} \end{array} \end{array}$$

The *discrete Haar wavelet transform* is the *N* × *N* matrix *𝒲* ^*N*^ : *𝕂* ^*N*^ ⊂ *ℓ* ^2^ → *𝕂* ^*N*^ ⊂ *ℓ* ^2^ which maps the vector

(54) $$\begin{array}{c} Y\equiv \left\{Y_{i}\right\},\;\left(i=0,\ldots ,2^{M}-1,\;2^{M}=N<\infty ,\;M\in \mathbb{N}\right) \end{array}$$

into the vector of *wavelet coefficients* **β**_*N*_ = {*α*, *β*_*k*_^*n*^}:

(55) $$\begin{array}{cc} & {𝒲}_{N}Y=\beta_{N},\\ & \beta_{N}\overset{\mathrm{def}}{=}\left\{\alpha ,\beta_{0}^{0},\ldots ,\beta_{2^{M-1}-1}^{M-1}\right\},\\ & Y\overset{\mathrm{def}}{=}\left\{Y_{0},Y_{1},\ldots ,Y_{N-1}\right\},\;\left(2^{M}=N\right). \end{array}$$

The matrix *𝒲*_*N*_ can be easily computed by some recursive product [3, 4, 13, 29, 46] so that with *N* = 4, *M* = 2, we have [3, 4, 29]

(56) $$\begin{array}{c} {𝒲}_{4}=\left(\begin{array}{cccc} \frac{1}{2} & \frac{1}{2} & \frac{1}{2} & \frac{1}{2}\\ -\frac{1}{2} & -\frac{1}{2} & \frac{1}{2} & \frac{1}{2}\\ -\frac{1}{\sqrt{2}} & \frac{1}{\sqrt{2}} & 0 & 0\\ 0 & 0 & -\frac{1}{\sqrt{2}} & \frac{1}{\sqrt{2}} \end{array}\right). \end{array}$$

From (55) with *M* = 2, *N* = 4, by explicit computation, we have

(57) $$\begin{array}{c} \alpha =\frac{1}{4}\left(Y_{0}+Y_{1}+Y_{2}+Y_{3}\right) \end{array}$$

and [1–3, 14]

(58) $$\begin{array}{cc} & \beta_{0}^{0}=\frac{1}{2}\left(Y_{2}-Y_{0}+Y_{3}-Y_{1}\right),\\ & \beta_{0}^{1}=\frac{1}{\sqrt{2}}\left(Y_{0}-Y_{1}\right),\\ & \beta_{1}^{1}=\frac{1}{\sqrt{2}}\left(Y_{3}-Y_{2}\right). \end{array}$$

Thus the first wavelet coefficient *α* represents the average value of the sequence and the other coefficients *β* the finite differences. The wavelet coefficients *β*'s, also called details coefficients, are strictly connected with the first-order properties of the discrete time series.

In the following we will consider the short wavelet transform which consists in the subdivision of the DNA sequence into 4-length segments and apply the wavelet transform to each segment. As a result, from the *N* = 2^*M*^-length complex vector **Y**, which is subdivided into 2^*M*−2^ segments, the 4-parameter short Haar wavelet transform gives the cluster of points

(59) $$\begin{array}{c} \left({𝒲}^{p}ℜ\left(Y^{s}\right),{𝒲}^{p}ℑ\left(Y^{s}\right)\right),\;s=0,\ldots ,\;\;\sigma =\frac{N}{p},\;\;p=4 \end{array}$$

in the 8-dimensional space ℝ^4^ × ℝ^4^, that is,

(60) $$\begin{array}{c} \left(\alpha ,\alpha^{*}\right),\left(\beta_{0}^{0},{\beta^{*}}_{0}^{0}\right),\ldots ,\left(\beta_{2^{p-1}-1}^{p-1},{\beta^{*}}_{2^{p-1}-1}^{p-1}\right),\;p=4 \end{array}.$$

This algorithm enables us to construct clusters of wavelet coefficients and to study the correlation between the real and imaginary coefficients of the DNA representation and DNA walk. It has been observed [3, 4, 29] that some symmetry arises from the plots of wavelet coefficients of DNA walks.

### 6.1. Cluster Analysis of the Wavelet Coefficients of the Complex DNA Representation

**Figure 13:**
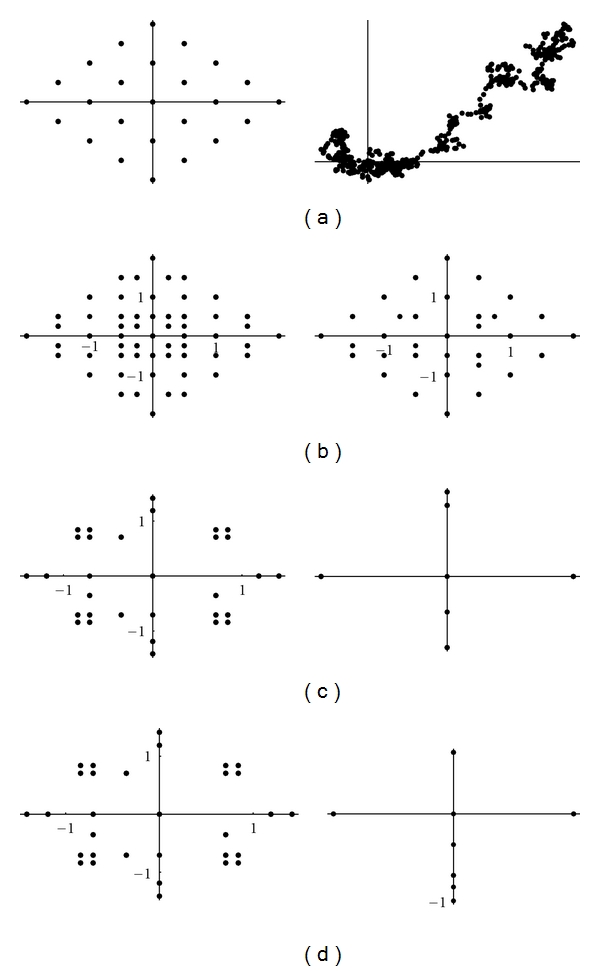
Cluster analysis of the 4th short Haar wavelet transform of a 4000-length random sequence (left) and its 2000-length random walk (right): (a) (*α*, α*); (b) (*β*_0_^0^, β*_0_^0^); (c) (*β*_0_^1^, β*_0_^1^); (d) (*β*_1_^1^, β*_1_^1^).

Let us first compute the clusters of wavelet coefficients for the random sequence (48). As can be seen the wavelet coefficients both for the sequence and for its series range in some discrete set of values (see Figure 13).

**Figure 14:**
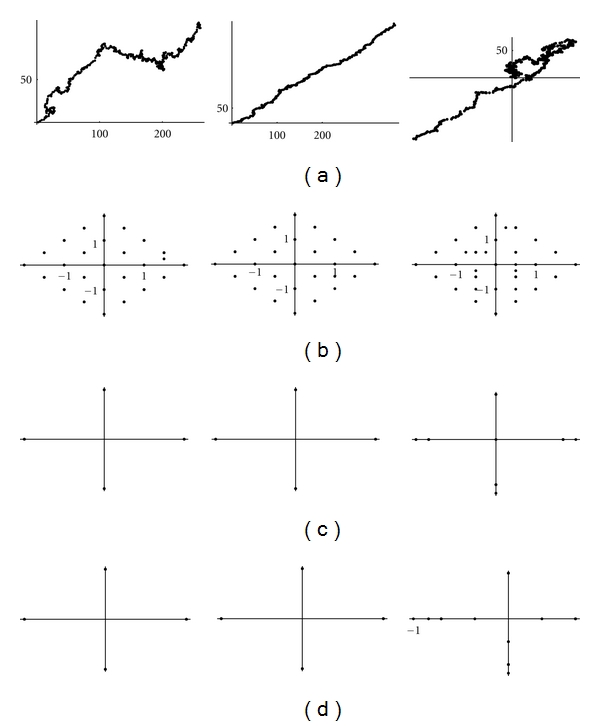
Cluster analysis of the 4th short Haar wavelet transform of the complex representation for a DNA walk on the first 2000 nucleotides of (**h1**) Aeropyrum, (**h2**) Acidianus, (**h3**) Acidilobus saccharovorans in the planes: (a) (*α*, α*); (b) (*β*_0_^0^, β*_0_^0^); (c) (*β*_0_^1^, β*_0_^1^); (d) (*β*_1_^1^, β*_1_^1^).

The cluster algorithm applied to the complex representation sequence shows that the values of the wavelet coefficients belong to some discrete finite sets (Figure 14).

**Figure 15:**
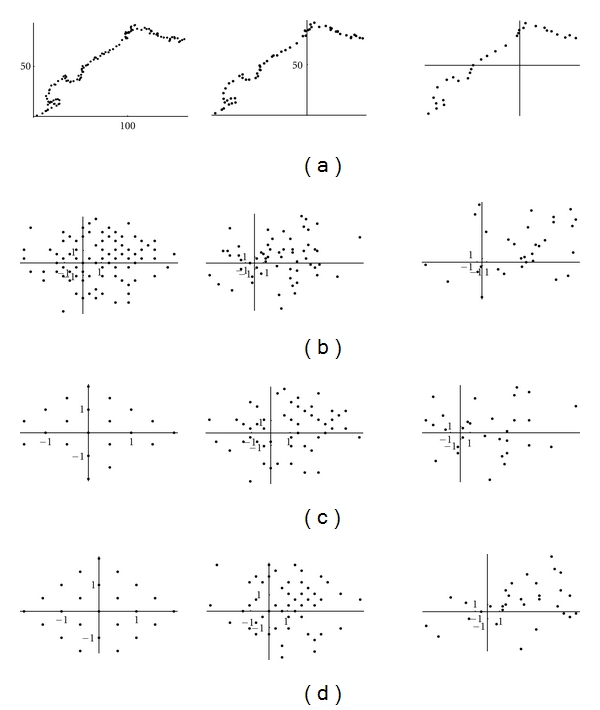
Cluster analysis of the 8th (left), 16th (middle column), 32th (right) short Haar wavelet transform of the DNA walk on the first 1000 nucleotides of h1 (Aeropyrum) in the planes: (a) (*α*, α*); (b) (*β*_0_^0^, β*_0_^0^); (c) (*β*_0_^1^, β*_0_^1^); (d) (*β*_1_^1^, β*_1_^1^).

**Figure 16:**
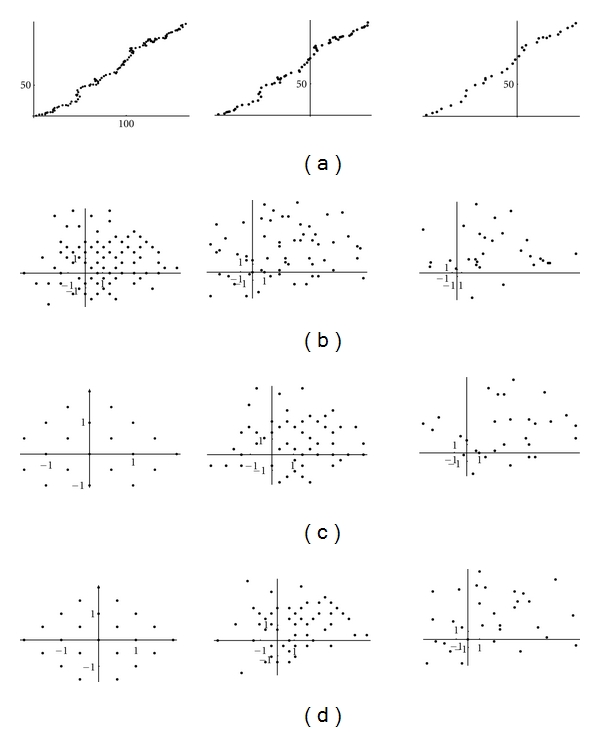
Cluster analysis of the 8th (left), 16th (middle column), 32th (right) short Haar wavelet transform of the DNA walk on the first 1000 nucleotides of h2 (Acidianus) in the planes: (a) (*α*, α*); (b) (*β*_0_^0^, β*_0_^0^); (c) (*β*_0_^1^, β*_0_^1^); (d) (*β*_1_^1^, β*_1_^1^).

**Figure 17:**
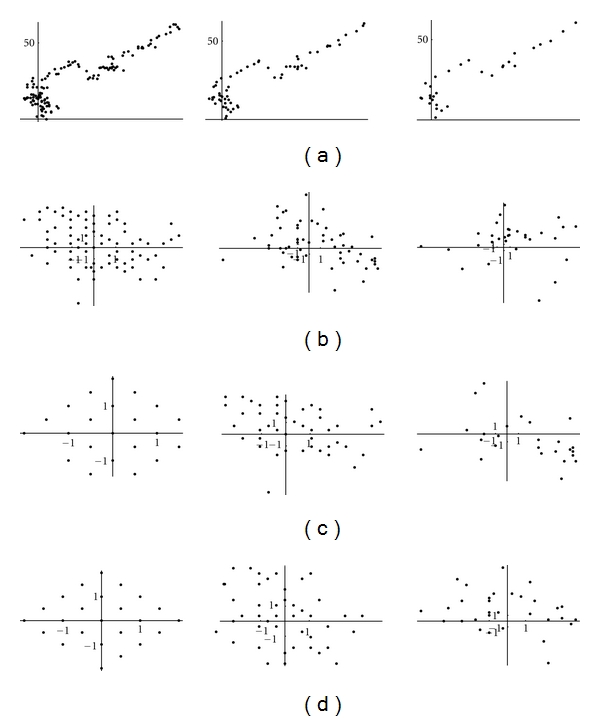
Cluster analysis of the 8th (left), 16th (middle column), 32th (right) short Haar wavelet transform of the DNA walk on the first 1000 nucleotides of h3 (Acidilobus saccharovorans) in the planes: (a) (*α*, α*); (b) (*β*_0_^0^, β*_0_^0^); (c) (*β*_0_^1^, β*_0_^1^); (d) (*β*_1_^1^, β*_1_^1^).

It should be noticed that this symmetry on detail coefficients is lost for wavelet transform on longer segments (Figures 15, 16 and 17).

There follows that DNA sequences have to be considered as Markov chain with short range dependence; in other words any acid nucleic is attached to the chain on the base of a correlation of the previous acid nucleic. In other words, if we look for a dependence rule on the DNA nucleotides this dependence might be summarized by a function as

(61) $$\begin{array}{c} x_{n+1}=f\left(x_{n}\right),\;\left(n=1,\ldots ,N\right). \end{array}$$

## 7. Conclusions

In this paper archaea DNAs have been studied by focussing on the main parameters for complexity. It has been shown that more or less the main indices for complexity and heterogeneity, such as entropy, fractal dimension, and complexity do not differ too much when we have to classify the complexity of the sequence. However, some DNA sequences look more close to random sequences than others, thus suggesting that the evolution involves a process of complexity reduction: the more evolved a sequence is, the more far from a random distribution it is. In any case seems to be apparently impossible to distinguish between a random sequence and a DNA chain. By using the short wavelet transform instead we have shown that on short range (4-nucleotides) a DNA sequence shows some symmetries that slowly disappear by increasing the length of the analysed segment. Moreover, more evolved organisms have a more symmetrical distribution of wavelet coefficients.

## Figures and Tables

**Table 1:** Correspondence codons to amino acids.

	Amino acid		Codon
1	M	Methionine	ATG
2	E	Glutamic acid	GAA, GAG
3	Q	Glutamine	CAA, CAG
4	D	Aspartic acid	GAT, GAC
5	R	Arginine	CGT, CGC, CGA, CGG, AGA, AGG
6	T	Threonine	ACT, ACC, ACA, ACG
7	N	Asparagine	AAT, AAC
8	H	Histidine	CAT, CAC
9	V	Valine	GTT, GTC, GTA, GTG
10	G	Glycine	GGT, GGC, GGA, GGG
11	L	Leucine	TTA, TTG, CTT, CTC, CTA, CTG
12	S	Serine	TCT, TCC, TCA, TCG, AGT, AGC
13	P	Proline	CCT, CCC, CCA, CCG
14	F	Phenylalanine	TTT, TTC
15	I	Isoleucine	ATT, ATC, ATA
16	C	Cysteine	TGT, TGC
17	A	Alanine	GCT, GCC, GCA, GCG
18	K	Lysine	AAA, AAG
19	Y	Thyroxine	TAT, TAC
20	W	Tryptophan	TGG
		Stop	TAA, TAG, TGA

**Table 2:** Randomness.

Mycoplasma putrefaciens	0.696					

Mortierella verticillata			0.779			

Blattabacterium		0.743				

*Aeropyrum pernix*					0.982	

*Acidianus hospitalis*			0.828			

Acidilobus saccharouorans				0.934		

pseudorandom						0.999

**Table 3:** Randomness of amino acids distribution.

Mycoplasma putrefaciens			0.946				

Mortierella verticillata		0.938					

*Blattabacterium*					0.953		

*Aeropyrum pernix*						0.962	

*Acidianus hospitalis*	0.916						

Acidilobus saccharouorans				0.950			

pseudorandom							0.963

**Table 4:** Complexity.

Mycoplasma putrefaciens	1.151						

Mortierella verticillata					1.285		

*Blattabacterium*		1.197					

*Aeropyrum pernix*			1.212				

*Acidianus hospitalis*				1.231			

Acidilobus saccharouorans							1.296

Pseudorandom						1.295	

**Table 5:** Fractal dimensions.

Mycoplasma putrefaciens	1.283							

Mortierella verticillata						1.296		

*Blattabacterium*			1.287					

*Aeropyrum pernix*				1.288				

*Acidianus hospitalis*					1.290			

Acidilobus saccharouorans							1.297	

pseudorandom								1.298

pseudoperiodic		1.285						

**Table 6:** Shannon entropy.

*Mycoplasma putrefaciens*	0.877					

*Mortierella verticillata*					0.976	

*Blattabacterium*		0.911				

*Aeropyrum pernix*			0.922			

*Acidianus hospitalis*				0.937		

*Acidilobus saccharovorans*						0.984

pseudorandom						0.984
